# Association of Prenatal Exposure to Early-Life Adversity With Neonatal Brain Volumes at Birth

**DOI:** 10.1001/jamanetworkopen.2022.7045

**Published:** 2022-04-12

**Authors:** Regina L. Triplett, Rachel E. Lean, Amisha Parikh, J. Philip Miller, Dimitrios Alexopoulos, Sydney Kaplan, Dominique Meyer, Christopher Adamson, Tara A. Smyser, Cynthia E. Rogers, Deanna M. Barch, Barbara Warner, Joan L. Luby, Christopher D. Smyser

**Affiliations:** 1 Department of Neurology, Washington University in St Louis, St Louis, Missouri; 2 Department of Psychiatry, Washington University in St Louis, St Louis, Missouri; 3 School of Medicine, Washington University in St Louis, St Louis, Missouri; 4 Department of Biostatistics, Washington University in St Louis, St Louis, Missouri; 5 Developmental Imaging, Murdoch Children’s Institute, Melbourne, Australia; 6 Electrical and Electronic Engineering, University of Melbourne, Melbourne, Australia; 7 Department of Pediatrics, Washington University in St Louis, St Louis, Missouri; 8 Department of Psychological and Brain Sciences, Washington University in St Louis, St Louis, Missouri; 9 Department of Radiology, Washington University in St Louis, St Louis, Missouri

## Abstract

**Importance:**

Exposure to early-life adversity alters the structural development of key brain regions underlying neurodevelopmental impairments. The association between prenatal exposure to adversity and brain structure at birth remains poorly understood.

**Objective:**

To examine whether prenatal exposure to maternal social disadvantage and psychosocial stress is associated with neonatal global and regional brain volumes and cortical folding.

**Design, Setting, and Participants:**

This prospective, longitudinal cohort study included 399 mother-infant dyads of sociodemographically diverse mothers recruited in the first or early second trimester of pregnancy and their infants, who underwent brain magnetic resonance imaging in the first weeks of life. Mothers were recruited from local obstetric clinics in St Louis, Missouri from September 1, 2017, to February 28, 2020.

**Exposures:**

Maternal social disadvantage and psychosocial stress in pregnancy.

**Main Outcomes and Measures:**

Confirmatory factor analyses were used to create latent constructs of maternal social disadvantage (income-to-needs ratio, Area Deprivation Index, Healthy Eating Index, educational level, and insurance status) and psychosocial stress (Perceived Stress Scale, Edinburgh Postnatal Depression Scale, Everyday Discrimination Scale, and Stress and Adversity Inventory). Neonatal cortical and subcortical gray matter, white matter, cerebellum, hippocampus, and amygdala volumes were generated using semiautomated, age-specific, segmentation pipelines.

**Results:**

A total of 280 mothers (mean [SD] age, 29.1 [5.3] years; 170 [60.7%] Black or African American, 100 [35.7%] White, and 10 [3.6%] other race or ethnicity) and their healthy, term-born infants (149 [53.2%] male; mean [SD] infant gestational age, 38.6 [1.0] weeks) were included in the analysis. After covariate adjustment and multiple comparisons correction, greater social disadvantage was associated with reduced cortical gray matter (unstandardized β = −2.0; 95% CI, −3.5 to −0.5; *P* = .01), subcortical gray matter (unstandardized β = −0.4; 95% CI, −0.7 to −0.2; *P* = .003), and white matter (unstandardized β = −5.5; 95% CI, −7.8 to −3.3; *P* < .001) volumes and cortical folding (unstandardized β = −0.03; 95% CI, −0.04 to −0.01; *P* < .001). Psychosocial stress showed no association with brain metrics. Although social disadvantage accounted for an additional 2.3% of the variance of the left hippocampus (unstandardized β = −0.03; 95% CI, −0.05 to −0.01), 2.3% of the right hippocampus (unstandardized β = −0.03; 95% CI, −0.05 to −0.01), 3.1% of the left amygdala (unstandardized β = −0.02; 95% CI, −0.03 to −0.01), and 2.9% of the right amygdala (unstandardized β = −0.02; 95% CI, −0.03 to −0.01), no regional effects were found after accounting for total brain volume.

**Conclusions and Relevance:**

In this baseline assessment of an ongoing cohort study, prenatal social disadvantage was associated with global reductions in brain volumes and cortical folding at birth. No regional specificity for the hippocampus or amygdala was detected. Results highlight that associations between poverty and brain development begin in utero and are evident early in life. These findings emphasize that preventive interventions that support fetal brain development should address parental socioeconomic hardships.

## Key Points

**Question:**

Is prenatal exposure to maternal social disadvantage and psychosocial stress associated with global and relative infant brain volumes at birth?

**Findings:**

In this longitudinal, observational cohort study of 280 mother-infant dyads, prenatal exposure to greater maternal social disadvantage, but not psychosocial stress, was associated with statistically significant reductions in white matter, cortical gray matter, and subcortical gray matter volumes and cortical folding at birth after accounting for maternal health and diet.

**Meaning:**

These findings suggest that prenatal exposure to social disadvantage is associated with global reductions in brain volumes and folding in the first weeks of life.

## Teaser

This cohort study of mother-infant dyads examines whether maternal social disadvantage and psychosocial stress are associated with neonatal brain volumes at birth.

## Introduction

Childhood exposure to early-life adversity (ELA), such as poverty, parental psychopathology, and psychosocial or physiological stress, is a well-described risk factor for adverse neurodevelopmental, socioemotional, and health outcomes.^1,2,3,4,5^ The pathways by which ELA is biologically embedded are complex and incompletely understood, with hypotheses centered on the effects of material deprivation, environmental exposures, and stressful psychosocial experiences on the hypothalamic-pituitary-adrenal (HPA) axis and systemic inflammation.^3,6,7,8^ Human and animal studies^2,7,9,10,11^ posit altered structural brain development as a key mechanism by which ELA contributes to poor outcomes. Magnetic resonance imaging (MRI) studies^12,13,14,15,16,17^ suggest that poverty in early childhood is associated with reduced cortical gray and white matter, hippocampus, and amygdala volumes at school age. In turn, reduced cortical and hippocampal volumes in childhood mediate associations between ELA (eg, poverty and family stress) and cognitive and behavioral impairments.^16,17,18,19^ Despite clear and compelling links between ELA and childhood neurodevelopment,^1,2,3,4^ much less is known about its prenatal effects.

The prenatal period is a particularly vulnerable stage of brain development,^20,21^ containing most neurogenesis and neuronal migration, with ongoing synaptogenesis, pruning, and myelination throughout the second and third trimesters.^22^ A small but growing body of literature demonstrates lasting consequences of prenatal exposure to ELA on childhood outcomes, including cognitive delays and externalizing disorders.^23,24,25^ However, few studies have explored the association between prenatal ELA and brain outcomes at birth, and cumulative or dimensional models have rarely been applied.^26^ The extant prenatal literature has largely conducted parallel lines of research concentrating on specific factors, including maternal alcohol or other substance use, health conditions, or psychosocial stress (ie, mood or affect problems, stress, and trauma).^27^ Few studies have examined prenatal exposure to poverty or multiple other factors,^26,27^ despite their overlapping findings.^28^

To date, studies^29,30,31^ investigating maternal perinatal psychosocial stress in association with neonatal brain volumes in healthy infants have focused on the hippocampus and amygdala, with differential findings for offspring sex, exposures, and the timing of those exposures. Maternal depression and/or stress during pregnancy were associated with altered hippocampus, amygdala, and cerebellum volumes and cortical folding in utero and shortly after birth.^30,31,32,33^ These studies reported negative associations between maternal psychosocial stress and income,^29,31^ but they represented populations of higher socioeconomic status (SES) and/or did not consistently control for SES.^32,33^ Although studies of early childhood SES also demonstrate consistent associations with hippocampus volume,^2,8^ limited fetal and neonatal MRI investigations have found an association between lower parental SES in pregnancy and global metrics, including altered cortical gray matter volumes,^34,35,36^ increased gyrification,^34^ and decreased white matter, deep gray matter, cerebellum, and brainstem volumes.^34,35,36^ Independent of maternal educational level, maternal smoking and psychiatric history in pregnancy have been found to explain variability in neonatal brain volumes.^36^

Given the US rates of childhood poverty (16%)^37^ and maternal perinatal mood disorders (14% for depression and 11%-20% for anxiety),^38^ prenatal ELA likely affects a significant proportion of the population. Furthermore, pregnant women with low incomes are at disproportionately greater risk of psychiatric disorders^39,40^ and stress during pregnancy.^41^ Consequently, it is essential to evaluate the contributions of psychosocial stress and poverty to in utero brain development in order to design preventive strategies.

We addressed this critical gap by quantifying prenatal exposures to latent constructs of maternal psychosocial stress (depression, stress, and lifetime interpersonal traumas or stressors) and social disadvantage (broad measure of SES and related factors) along with maternal health, tobacco use, and marijuana exposure in healthy, term-born infants. We investigated the associations between these factors and neonatal brain volumes at birth (global measures of cortical and subcortical gray matter, white matter, and cerebellar volume and cortical folding) along with 2 structures of interest (amygdala and hippocampus). On the basis of existing literature, we hypothesized that greater maternal social disadvantage and psychosocial stress would each be independently associated with lower neonatal brain volumes and reduced cortical folding. Given the sensitivity of subcortical structures to HPA axis activation,^8,42,43^ we expected to observe regionally specific susceptibility of the hippocampus and amygdala to social disadvantage and psychosocial stress exposure.

## Methods

### Study Design and Population

In this longitudinal, observational, multiwave, multimethod collaboration, a cohort of pregnant women who participated in a large-scale study of preterm birth^44^ within the Washington University in St Louis March of Dimes Prematurity Research Center were recruited from September 1, 2017, to February 28, 2020. Women from the parent study (n = 663) were invited to participate in this investigation (see Luby et al^45^ for cohort details) with the following exclusion criteria: multiple gestation, infections known to cause congenital disease (eg, syphilis), and/or alcohol or drug use other than tobacco and marijuana. A total of 395 eligible participating mothers completed assessments during each trimester of pregnancy and at delivery. Medical data from mothers and their 399 singleton offspring (4 mothers had 2 singleton births during the recruitment period) were collected from questionnaires and medical record review. In order to assess the contributions of racial and ethnic discrimination and inequities, pregnant mothers’ self-reported race and ethnicity were extracted from the medical record. The following options were provided for race: American Indian/Alaskan Native, Asian, Black or African American, Native Hawaiian/Pacific Islander, White, unknown, or other (free text), and the following options for ethnicity: Hispanic/Latina, non-Hispanic/Latina, or unknown/not applicable. Neonatal brain MRI was performed in the first weeks of life only on infants born before the COVID-19 pandemic. Exclusion criteria included premature birth (<37 weeks’ gestation), neonatal intensive care unit admission for more than 7 days, birth weight less than 2000 g, or evidence of brain injury on MRI. After exclusion and data quality criteria were applied, 280 mother-infant dyads were included in current analysis (eFigure in the Supplement). Study procedures were reviewed and approved by the Washington University Institutional Review Board. Written informed consent was obtained for each participant, with written parental informed consent for each infant. The study followed the Strengthening the Reporting of Observational Studies in Epidemiology (STROBE) reporting guideline for cohort studies.^46^

### Measures

#### Maternal Social Disadvantage and Psychosocial Stress

Confirmatory factor analysis was used to derive 2 latent maternal social disadvantage and maternal psychosocial stress constructs.^45^ The following maternal measures were included in the social disadvantage construct: health insurance status (grouped by private insurance or public or no insurance), highest educational level, income-to-needs ratio^47^ in each trimester, national Area Deprivation Index percentile at birth,^48^ and Healthy Eating Index.^49^ The following maternal psychological measures were included in the psychosocial stress construct: Perceived Stress Scale^50^ and Edinburgh Postnatal Depression Scale (EPDS)^51^ in each trimester, Stress and Adversity Inventory,^52^ and Everyday Discrimination Scale (eMethods in the Supplement).^53^

#### Maternal Comorbidities and Exposures

A maternal medical risk score was calculated for each participant using questionnaires and medical record review.^54^ This validated index^55^ is a sum of weighted comorbidities, including advanced age, cardiac disease, and preeclampsia, with higher scores predicting increased risk of severe morbidity or mortality. Frequency of tobacco and marijuana use (none, some, or heavy) (Table 1) was self-reported on questionnaires at each trimester. At the discretion of the treating clinician, a subset of mothers underwent urine drug screens during prenatal clinical care. Marijuana exposure (any vs none) was, therefore, based on self-report and/or a urine drug screen result positive for tetrahydrocannabinol metabolites. Because maternal prepregnancy body mass index, marijuana exposure, and tobacco use are not included in the maternal medical risk index, they were independently evaluated as covariates of interest.

**Table 1.** Social Background and Infant Clinical Characteristics of the Sample

Characteristic	Data (N = 280)
Maternal age, mean (SD) [range], y	29.1 (5.3) [18.7 to 41.8]
Maternal race and ethnicity (self-identified), No. (%)	
Black/African American	170 (60.7)
White	100 (35.7)
Other^a^	10 (3.6)
Maternal medical risk score, median (IQR) [range]	1.0 (0.0 to 2.0) [0 to 8]
Self-reported maternal tobacco use, No. (%)	
Heavy use (≥6 cigarettes daily)	16 (5.7)
Some use (<6 cigarettes daily)	20 (7.1)
None	244 (87.1)
Any maternal marijuana exposure, No. (%)	74 (26.4)
Positive urine drug screen result, No. (%)^b^	59 (21.1)
Self-reported maternal marijuana use, No. (%)	
Daily use	21 (7.5)
Some use (less than daily)	15 (5.4)
None	244 (87.1)
Insurance, No. (%)	
Medicaid or Medicare	105 (37.6)
Individual or group health insurance	144 (51.4)
Uninsured	31 (11.0)
Married mothers, No. (%)	99 (35.4)
Maternal educational level (n = 272), No. (%)	
Did not complete high school	28 (10.3)
Finished high school or GED	68 (25.0)
Some college or vocational school	83 (30.5)
College degree (4 y)	34 (12.5)
Graduate degree	59 (21.7)
Income-to-needs ratio, median (IQR) [range]	
Trimester	
First (n = 271)	1.25 (0.89 to 3.80) [0.43 to 12.15]
Second (n = 216)	1.65 (0.91 to 5.17) [0.38 to 12.15]
Third (n = 238)	1.46 (0.89 to 5.17) [0.35 to 11.83]
Area Deprivation Index score, mean (SD) [range]	68.2 (24.9) [1 to 100]
Healthy Eating Index score (n = 223), mean (SD) [range]	58.8 (10.0) [33.0 to 80.7]
Social disadvantage, mean (SD) [range]	–0.04 (0.97) [–2.2 to 1.5]
Perceived Stress Scale score, mean (SD) [range]	
Trimester	
First (n = 276)	13.1 (7.2) [0 to 35]
Second (n = 215)	12.9 (7.5) [0 to 36]
Third (n = 234)	12.5 (7.3) [0 to 37]
Edinburgh Postpartum Depression Scale score, median (IQR) [range]	
Trimester	
First (n = 278)	4.0 (1.0 to 7.0) [0 to 25]
Second (n = 235)	3.0 (1.0 to 7.0) [0 to 20]
Third (n = 239)	3.0 (1.0 to 6.0) [0 to 25]
STRAIN (n = 263), median (IQR) [range]	
Stressful event count	6.0 (3.0 to 11.0) [0 to 30]
Weighted severity	15.0 (7.0 to 29.0) [0 to 99]
Everyday Discrimination Scale score (n = 261), median (IQR) [range]^c^	1.0 (1.0 to 1.8) [1 to 6]
Psychosocial stress, mean (SD) [range]	–.11 (.88) [–1.7 to 3.7]
Infant gestational age, mean (SD) [range], wk	38.6 (1.0) [37 to 41]
Postmenstrual age at MRI, mean (SD) [range], wk	41.7 (1.3) [38 to 45]
Infant sex (male), No. (%)	149 (53.2)
Infant birth weight, mean (SD) [range], g	3257.7 (487.7) [2200 to 4627]

Abbreviation: STRAIN, Stress and Adversity Inventory.

^a^ Other includes Asian (n = 5), Latina (n = 3), Middle Eastern (n = 1), and Asian and White (n = 1).

^b^ A total of 119 mothers (42.5%) had urine drug screen data during pregnancy.

^c^ Everyday Discrimination Scale was scored for experiences of racial discrimination only (otherwise coded as 0).

#### MRI Data Collection, Preprocessing, and Volumetric Measures

All MRIs were performed within the first weeks of life without sedation during natural sleep. Magnetic resonance imaging data were collected using a Prisma 3T scanner and 64-channel head coil (Siemens). Infants (n = 10) without high-quality (ie, low motion) structural data as determined by an imaging scientist (D.A.) and pediatric neurologist (C.D.S.) were excluded. The Melbourne Children’s Regional Infant Brain Atlas Surface segmentation and surface extraction toolkit was used to generate segmentations into white and gray matter, cerebellum, brainstem, and subcortical gray matter and surface-based cortical parcellations from preprocessed T2-weighted images.^56,57^ See the eMethods in the Supplement for sequence parameters, preprocessing, and analysis procedures.

Brain volumes of interest included total cortical and subcortical gray matter, white matter, and cerebellum, in addition to right and left hippocampi and amygdalae. Total raw volumes for all structures were analyzed, along with standardized regional volumes for the hippocampi and amygdalae generated by dividing by total brain volume, as is common in neonatal neuroimaging studies.^30,33^ Cortical folding was measured using the total Gyrification Index (GI), a ratio of the cortical surface area divided by the cortical hull surface area.^58^

### Statistical Analysis

Analyses were performed using SPSS software, version 28 (IBM Corporation). Potential covariates were explored using Pearson correlation and 2-tailed, unpaired *t* tests. Maternal tobacco use, infant sex, birth weight, and postmenstrual age (PMA) at MRI were associated with brain volumes of interest (eTable 1 in the Supplement). These covariates and social disadvantage and psychosocial stress factor scores were included as independent variables in hierarchical linear regression analyses, each with brain volumes or cortical folding as the dependent variable. For each volume of interest, the first step accounted for maternal tobacco use (no use = 0), infant sex (female = 0), birth weight, and PMA at MRI. The social disadvantage and psychosocial stress factors were entered simultaneously in the second step of the model to determine the unique, independent proportion of variance (change in *R*^2^) explained in brain volume and folding outcomes over and above covariate factors. Regression models were checked for linearity, homoscedasticity, and absence of multicollinearity, and the residuals approximated a normal distribution. Results for primary outcomes were corrected for multiple comparisons using the Benjamini-Hochberg false discovery rate procedure.^59^ *P *values and false discovery rate–adjusted *P* values <.05 were considered to be statistically significant.

## Results

### Infant Characteristics

A total of 280 mothers (mean [SD] age, 29.1 [5.3] years; 170 [60.7%] Black/African American, 100 [35.7%] White, and 10 [3.6%] of other race or ethnicity) and their healthy, term-born infants (149 [53.2%] male; mean [SD] infant gestational age, 38.6 [1.0] weeks) were included in the study (Table 1). Male infants had a larger mean (SD) birth weight (3316 [470] g) than female infants (3191 [500] g) (*P* = .03) (eTable 2 in the Supplement). No sex differences were found for PMA at MRI, social disadvantage, and psychosocial stress (eTable 2 in the Supplement). At the time of MRI, infants had a mean (SD) PMA of 42.0 (1.3) weeks, which was slightly younger than infants excluded because of low-quality or missing MRI data. No other differences were found between the 2 groups (eTable 3 in the Supplement).

### Prenatal Life Adversity

Table 1 summarizes the prenatal life adversity characteristics of the sample, including the latent constructs of maternal social disadvantage and psychosocial stress. A total of 136 mothers (48.6%) in the cohort had public insurance or no health insurance. Median income-to-needs ratios at each trimester ranged from 1.25 to 1.65 (minimum, 0.38; maximum, 12.15). The median EPDS scores at each trimester ranged from 3.0 to 4.0 (minimum, 0; maximum, 25). Social disadvantage was correlated with more maternal psychosocial stress (*r* = 0.43, *P* < .001). Differences between this full-term cohort and the full sample (from which the factors were derived)^45^ were predominantly driven by infants born prematurely (eTable 4 in the Supplement).

### MRI Measures

#### Brain Volumes

**Figure 1.**
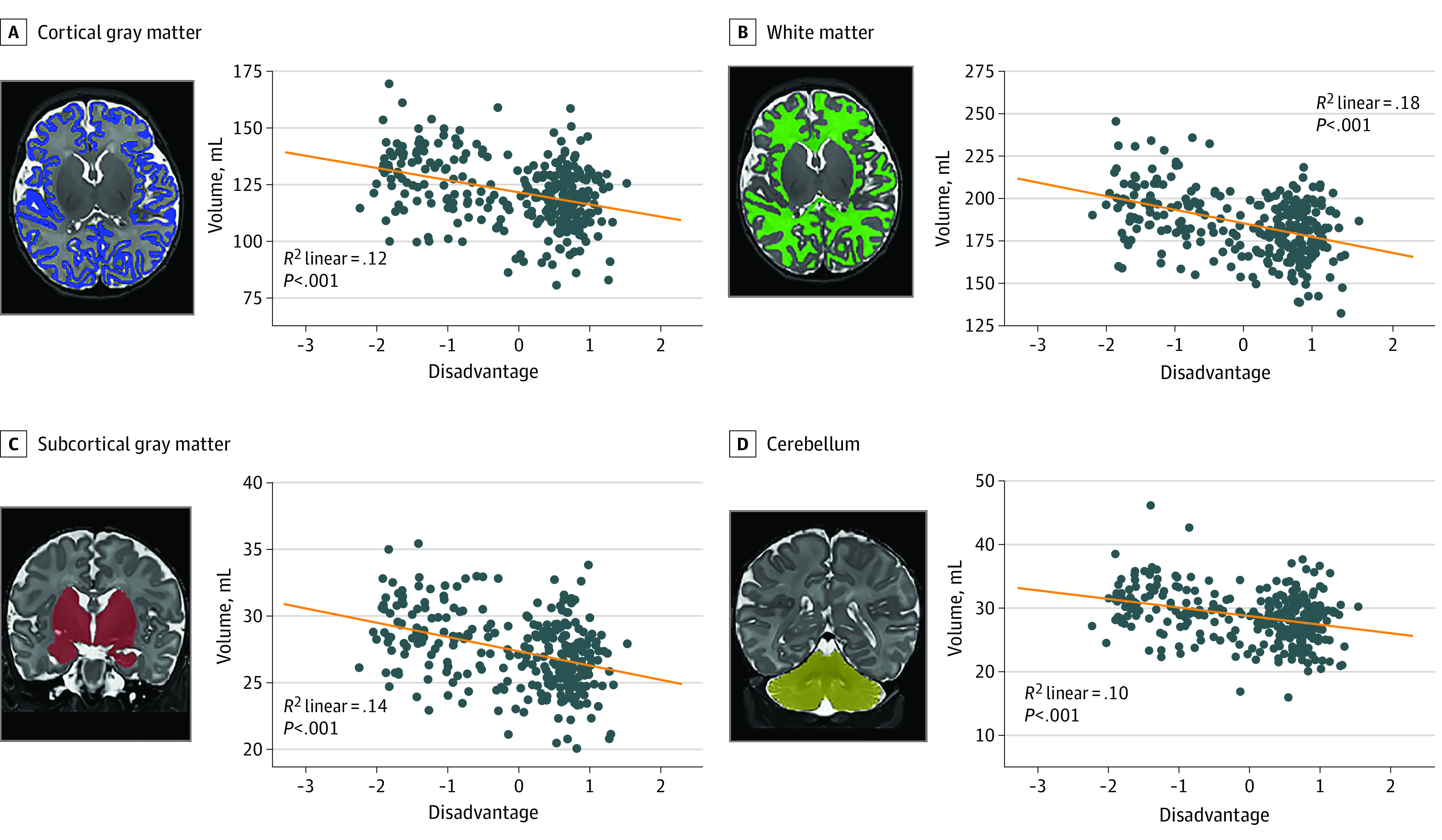
Correlation Between Total Brain Volume and Maternal Social Disadvantage Factor Correlation and *P* values are included for line of best fit. Automated volumetric segmentation for each tissue type is overlaid on T2-weighted image for a representative infant. X-axis indicates maternal social disadvantage.

Table 2 summarizes the second, final step of the hierarchical linear regression results (full results in eTable 5 in the Supplement). In step 1, female sex, lower birth weight, and younger PMA at MRI were associated with smaller cortical (sex: β = 0.23, *P* < .001; birth weight: β = 0.29, *P* < .001; and PMA at MRI: β = 0.54, *P* < .001) and subcortical gray matter (sex: β = 0.23, *P* < .001; birth weight: β = 0.25, *P* < .001; and PMA at MRI: β = 0.54, *P* < .001), white matter (sex: β = 0.28, *P* < .001; birth weight: β = 0.27, *P* < .001; and PMA at MRI: β = 0.22, *P* < .001), and cerebellar (sex: β = 0.23, *P* < .001; birth weight: β = 0.21, *P* < .001; and PMA at MRI: β = 0.62, *P* < .001) volumes (eTable 5 in the Supplement). Tobacco use was associated with reduced subcortical gray (β = −0.11, *P* = .01) and white matter (β = −0.12, *P* = .02) (eTable 5 in the Supplement). In step 2, greater social disadvantage was associated with reduced volumes across all tissue types (Table 2 and Figure 1), except for the cerebellum (eTable 5 in the Supplement). Social disadvantage accounted for an additional 1.6% of the variance for total cortical gray matter (unstandardized β = –2.0; 95% CI, –3.5 to –0.5), 2.6% for subcortical gray matter (unstandardized β = –0.4; 95% CI, –0.7 to –0.2), and 7% for white matter (unstandardized β = –5.5; 95% CI, –7.8 to –3.3) (eTable 5 in the Supplement). The contribution of psychosocial stress was not significant (Table 2). A similar pattern of results was found for total brain volume (eTable 6 in the Supplement). Post hoc analyses showed similar results for the left and right hemispheric cortical gray matter, cerebral white matter, and cerebellar hemispheres (eTable 7 in the Supplement).

**Table 2.** Summary of Final Step in Hierarchical Linear Regression Assessing the Association of Maternal Social Disadvantage and Psychosocial Stress With Structural MRI Measures at Birth^a^

Variable	Standardized β	*P* value	*Q* value^b^
Total cortical gray matter (*R*^2^ = .56, *P* < .001)			
Sex	0.24	<.001	<.001
Birth weight	0.24	<.001	<.001
PMA at MRI	0.52	<.001	<.001
Tobacco use	−0.03	.52	.69
Social disadvantage	−0.13	.008	.01
Psychosocial stress	−0.02	.59	.64
Total subcortical gray matter (*R*^2^ = .56, *P* < .001)			
Sex	0.24	<.001	<.001
Birth weight	0.20	<.001	<.001
PMA at MRI	0.52	<.001	<.001
Tobacco use	−0.06	.17	.67
Social disadvantage	−0.16	.002	.003
Psychosocial stress	−0.05	.30	.60
Total white matter (*R*^2^ = .36, *P* < .001)			
Sex	0.29	<.001	<.001
Birth weight	0.18	<.001	.001
PMA at MRI	0.19	<.001	<.001
Tobacco use	−0.05	.34	.69
Social disadvantage	−0.28	<.001	<.001
Psychosocial stress	−0.03	.64	.64
Left hippocampus (*R*^2^ = .22, *P* < .001)			
Sex	0.16	.003	.008
Birth weight	0.11	.06	.06
PMA at MRI	0.29	<.001	<.001
Tobacco use	−0.03	.57	.70
Social disadvantage	−0.18	.007	.01
Psychosocial stress	0.02	.75	.93
Right hippocampus (*R*^2^ = .22, *P* < .001)			
Sex	0.14	.01	.02
Birth weight	0.14	.01	.02
PMA at MRI	0.26	<.001	<.001
Tobacco use	−0.06	.29	.58
Social disadvantage	−0.18	.007	.01
Psychosocial stress	0.01	.82	.93
Left amygdala (*R*^2^ = .41, *P* < .001)			
Sex	0.30	<.001	<.001
Birth weight	0.13	.01	.02
PMA at MRI	0.37	<.001	<.001
Tobacco use	−0.08	.09	.58
Social disadvantage	−0.20	<.001	.003
Psychosocial stress	0.005	.92	.93
Right amygdala (*R*^2^ = .42, *P* < .001)			
Sex	0.28	<.001	<.001
Birth weight	0.17	<.001	.003
PMA at MRI	0.38	<.001	<.001
Tobacco use	−0.06	.25	.58
Social disadvantage	−0.19	<.001	.003
Psychosocial stress	0.005	.93	.93
Gyrification index (*R*^2^ = .31, *P* < .001)			
Sex	0.12	.03	.03
Birth weight	0.10	.07	.07
PMA at MRI	0.40	<.001	<.001
Tobacco use	0.10	.07	.07
Social disadvantage	−0.26	<.001	<.001
Psychosocial stress	0.04	.46	.46

Abbreviations: MRI, magnetic resonance imaging; PMA, postmenstrual age.

^a^ Results for all steps of hierarchical linear regression are given in full in eTable 5 in the Supplement.

^b^ *Q* values represent *P* values after correction for multiple comparisons using the Benjamini-Hochberg false discovery rate procedure.

#### Hippocampus and Amygdala

**Figure 2.**
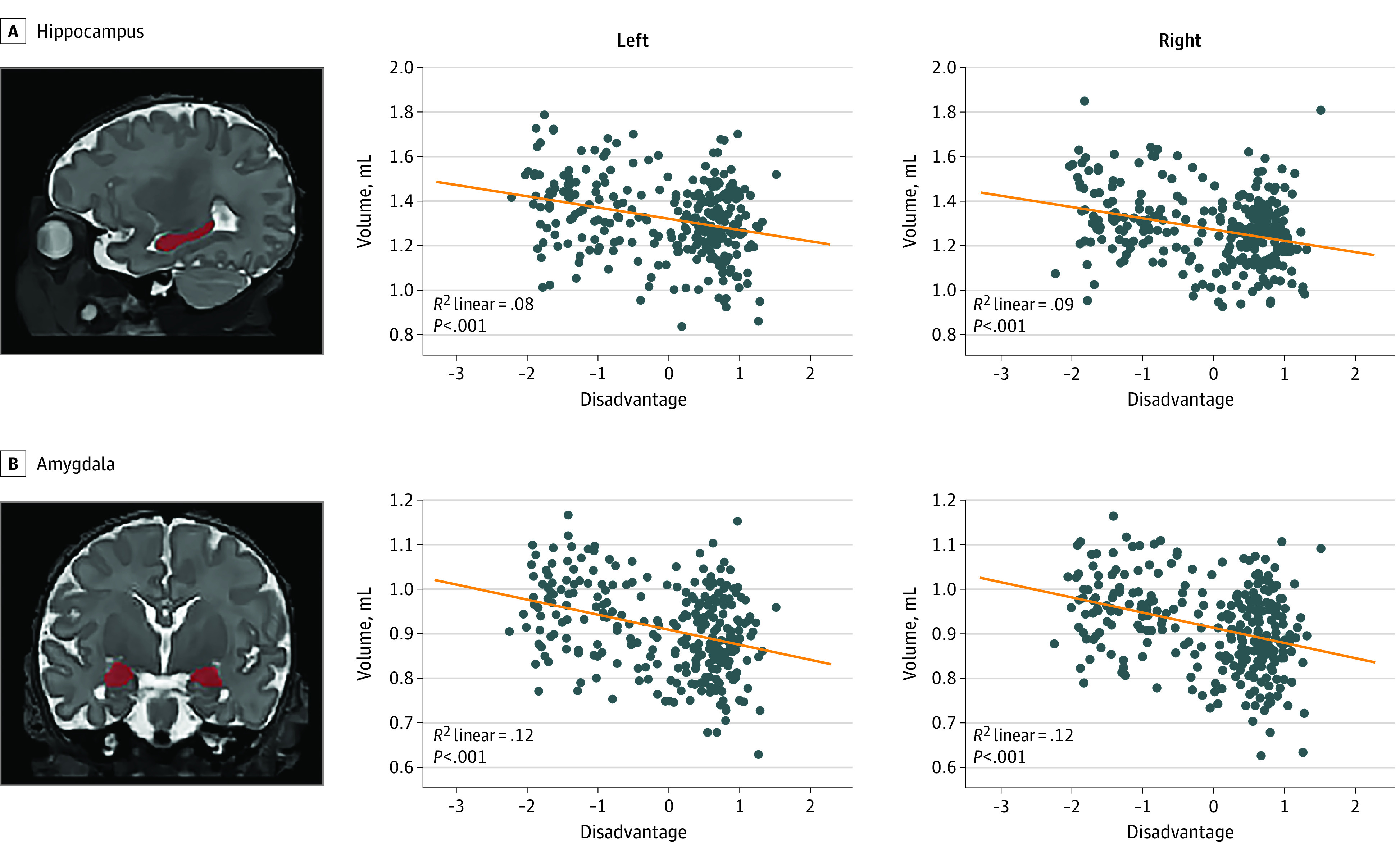
Correlation Between Regional Brain Volume and Maternal Social Disadvantage Factor Correlation and *P* values are included for line of best fit. Automated volumetric segmentation for each structure is overlaid on T2-weighted image for a representative infant. Note similar results across hemispheres. X-axis indicates maternal social disadvantage.

In step 1, female sex, lower birth weight, and younger PMA at MRI were associated with smaller right hippocampus (sex: β = 0.13, *P* = .02; birth weight: β = 0.20, *P* < .001; and PMA at MRI: β = 0.28, *P* < .001), left hippocampus (sex: β = 0.15, *P* = .006; birth weight: β = 0.16, *P* = .005; and PMA at MRI: β = 0.31, *P* < .001), right amygdala (sex: β = 0.27, *P* < .001; birth weight: β = 0.23, *P* < .001; and PMA at MRI: β = 0.40, *P* < .001) volumes, and left amygdala (sex: β = 0.29, *P* < .001; birth weight: β = 0.18, *P* < .001; and PMA at MRI: β = 0.39, *P* < .001) volumes (eTable 5 in the Supplement). Tobacco use was associated with reduced amygdalae volumes bilaterally (left amygdala: β = −0.13, *P* = .007; right amygdala: β = −0.11, *P* = .03) (eTable 5 in the Supplement). In step 2, greater social disadvantage was associated with reduced volumes for subcortical regions of interest and accounted for an additional 2.3% to 3.1% of the variance (Table 2 and Figure 2). Social disadvantage accounted for an additional 2.3% of the variance of the left hippocampus (unstandardized β = –0.03; 95% CI, –0.05 to –0.01), 2.3% of the right hippocampus (unstandardized β = –0.03; 95% CI, –0.05 to –0.01), 3.1% of the left amygdala (unstandardized β = –0.02; 95% CI, –0.03 to –0.01), and 2.9% of the right amygdala (unstandardized β = –0.02; 95% CI, –0.03 to –0.01) (eTable 5 in the Supplement). The contribution of psychosocial stress was not significant (Table 2). After standardization of hippocampal and amygdalae volumes using total brain volume, no significant associations were found with any covariates, social disadvantage, or psychosocial stress (eTable 5 in the Supplement).

#### Cortical Folding

**Figure 3.**
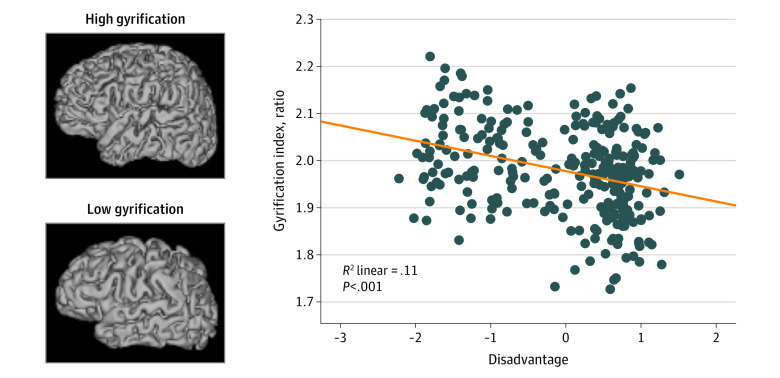
Correlation Between the Gyrification Index and Maternal Social Disadvantage Factor Cortical surfaces for representative infants with high vs low gyrification index are included for reference. X-axis indicates maternal social disadvantage.

In step 1, female sex, smaller birth weight, and younger PMA at MRI were associated with diminished GI (sex: β = 0.10, *P* = .05; birth weight: β = 0.17, *P* = .002; PMA at MRI: β = 0.43, *P* < .001) (eTable 5 in the Supplement). In step 2, higher social disadvantage was associated with reduced GI (β = −0.26, *P* < .001) and accounted for an additional 4.8% of the variance (unstandardized β = –0.03; 95% CI, –0.04 to –0.01) (Table 2 and Figure 3). Tobacco use and psychosocial stress were not significantly associated with cortical folding.

## Discussion

This cohort study is one of the largest investigations of the fetal origins of health and disease beginning in the first trimester of gestation using comprehensive, multidimensional measures of maternal social disadvantage and psychosocial stress to assess associations with brain morphometry at birth. In healthy, term-born infants, prenatal exposure to social disadvantage demonstrated inverse associations with all brain tissue types, including reduced cortical and subcortical gray and white matter and decreased cortical folding in the first weeks of life. After accounting for global differences in brain volume, no regionally specific associations were found between social disadvantage or psychosocial stress and the hippocampus and amygdala. In our cohort, exposure to greater social disadvantage in utero appeared to play a greater role in brain structural development than maternal psychosocial stress.

We provide evidence of the association of prenatal exposure to social disadvantage with differences in global brain structural development at birth. Results persisted after accounting for infant birth weight, which also is associated with SES.^60^ Likely because of rigorous covariate control, effect sizes were small but consistent with reports in other samples of infants^35^ and children.^16^ Furthermore, findings are consistent with cross-sectional studies that found that a lower income-to-needs ratio was associated with reduced total cortical and subcortical gray matter in infants at 5 weeks^35^ and 5 months of age.^13^ Findings also align with work that reported regional and widespread reductions in cortical folding associated with lower SES among older children.^14,61^ Of note, we extend prior work^13,14,25,61^ to show that the associations between poverty and reduced brain volumes begin in utero and are evident in the first weeks of life. Social disadvantage was most strongly associated with reduced white matter volume, explaining 7% of the variance. This finding highlights the timing of prenatal exposure to poverty and the vulnerability of white matter as myelination occurs rapidly beginning at 28 to 29 weeks of gestation.^62,63^ During fetal development, oligodendrocyte progenitor cells and subplate neurons are sensitive to oxidative stress, which may have cascading effects on pruning and/or crossing fibers and subsequent white matter volume at birth.^22,64^

Although greater social disadvantage during pregnancy was associated with global reductions in infant brain volume and cortical folding, the amygdalae and hippocampi were not preferentially associated with social disadvantage or psychosocial stress. Differences between our findings and studies reporting on the effects of poverty on these subcortical structures may be attributed to prior works^13,32,35^ relying on single measures of SES, assessing brain development at later time points, and/or including higher SES samples. We interpret current study findings as evidence of a more widespread alteration in brain growth and development in the setting of exposure to significant, multifactorial socioeconomic disadvantage in utero.

This study addresses the independent contributions of maternal SES and psychosocial stress during pregnancy on offspring brain morphometry at birth.^65^ Consistent with other findings,^66^ our measure of social disadvantage correlated with psychosocial stress during pregnancy. However, prenatal exposure to social disadvantage was associated with brain volumes and cortical folding, whereas psychosocial stress was not significant. Current results could reflect the fact that participants were oversampled for mothers with greater social disadvantage. We also assessed multiple aspects of social adversity, which when examined together are likely more impactful.^67,68^ We anticipate our results will be generalizable to other socioeconomically diverse (but otherwise relatively healthy) US populations. Results may not generalize to populations that face different kinds of adversity or those with higher SES.

Although the precise mechanism remains unclear, postnatal ELA studies^11,69^ posit that long-term deprivation of resources and/or psychosocial stress overstimulate the HPA axis and the immune system, leading to altered brain-behavior outcomes. Fetal sensitivity to glucocorticoids is a leading hypothesis to explain the regional effects of prenatal ELA on the hippocampus, amygdala, and prefrontal cortex.^42,43,70,71,72^ In addition, changes in maternal immune activation incited by prenatal ELA may contribute globally to brain development in utero via several mechanisms, including increased synaptic pruning, altered neurotransmitter profiles, impaired placental delivery of neurotrophic factors, and placental epigenetic programming.^73,74^

Through the above mechanisms, including changes in cortisol production and systemic inflammation, poverty and psychosocial stress likely have overlapping effects on the developing brain.^3,11,75,76^ Additional contributing factors for mothers living in poverty may include specific macronutrient and micronutrient deficiencies^77^ and direct neurotoxic and indirect neuroinflammatory effects of household, outdoor, and water pollutants, such as lead^78^ or air pollution.^79^ Future directions to elucidate causal mechanisms of neurodevelopmental and socioemotional impairments include examining specific maternal factors, such as inflammatory cytokines and cortisol,^42,80^ in the context of maternal psychological stress, SES, and related nutritional and environmental exposures. There is further work to be done to clearly establish links between prenatal ELA, brain morphometry findings, and childhood outcomes.^81,82^

### Limitations

Our findings should be interpreted in light of some study limitations. First, we assessed maternal depression with the EPDS. Although the EPDS is a validated measure, the lack of a semistructured interview may have led to symptom underreporting. Second, this study did not assess other environmental exposures, such as lead and air pollution, which may be linked with poverty and subsequent brain development. Third, we did not investigate the role of race in this analysis because of the collinearity between race and social disadvantage.^45^ This sample reflects the clear link between racial inequities and social disadvantage in the US and provides justification for including a measure of racial discrimination.

## Conclusions

In this cohort study, we examined the independent roles of maternal social disadvantage and psychosocial stress during pregnancy and found global associations between social disadvantage and neonatal brain volumetric and folding measures. No association was found between brain volumes and psychosocial stress. Of note, results highlight that associations between poverty and neurodevelopment begin in utero and are evident in the first weeks of life. These findings may inform future randomized clinical trials of poverty reduction and family-based interventions to address the material and psychosocial needs of expectant parents and improve neonatal brain outcomes at birth.^83^

## Notes

Supplement. **eFigure.** Participant Flow From Study Enrollment to Inclusion in Current Analysis **eMethods.** Supplemental Methods **eTable 1.** Identification of Covariates of Interest Associated With Neonatal Volumetric MRI Measures at Birth (N=280) **eTable 2.** Identification of Potential Covariates of Interest Associated With Infant Sex (N=280) **eTable 3.** Comparison of Full-term Infants Excluded Due to Missing/Low-Quality MRI Data **eTable 4.** Comparison of Full Cohort and Infants Excluded (Largely Due to Prematurity) From the Current Study **eTable 5.** Full Results of Hierarchical Linear Regression Linking Maternal Social Disadvantage and Psychosocial Stress With Structural MRI Measures at Birth (N=280) **eTable 6.** Hierarchical Linear Regression Linking Maternal Social Disadvantage and Psychosocial Stress with Total Brain Volumes (TBV) at Birth (N=280) **eTable 7.** Hierarchical Linear Regression Exploring Hemispheric Effects of Maternal Social Disadvantage and Psychosocial Stress (N=280)
