# Supplementary material for: Association of Prenatal Exposure to Early-Life Adversity With Neonatal Brain Volumes at Birth
Source: JAMA Netw Open. 2022 Apr 12;5(4):e227045. doi: 10.1001/jamanetworkopen.2022.7045 (PMC9006107; doi:10.1001/jamanetworkopen.2022.7045)
Supplement: Supplement. — eFigure. Participant Flow From Study Enrollment to Inclusion in Current Analysis eMethods. Supplemental Methods eTable 1. Identification of Covariates of Interest Associated With Neonatal Volumetric MRI Measures at Birth (N=280) eTable 2. Identification of Potential Covariates of Interest Associated With Infant Sex (N=280) eTable 3. Comparison of Full-term Infants Excluded Due to Missing/Low-Quality MRI Data eTable 4. Comparison of Full Cohort and Infants Excluded (Largely Due to Prematurity) From the Current Study eTable 5. Full Results of Hierarchical Linear Regression Linking Maternal Social Disadvantage and Psychosocial Stress With Structural MRI Measures at Birth (N=280) eTable 6. Hierarchical Linear Regression Linking Maternal Social Disadvantage and Psychosocial Stress with Total Brain Volumes (TBV) at Birth (N=280) eTable 7. Hierarchical Linear Regression Exploring Hemispheric Effects of Maternal Social Disadvantage and Psychosocial Stress (N=280) [file jamanetwopen-e227045-s001.pdf]

Supplementary Online Content

Triplett RL, Lean RE, Parikh A, et al. Association of prenatal exposure to early-life adversity with neonatal brain volumes at birth. *JAMA Netw Open*. 2022;5(4):e227045. doi:10.1001/jamanetworkopen.2022.7045

eFigure. Participant Flow From Study Enrollment to Inclusion in Current Analysis

eMethods. Supplemental Methods

eTable 1. Identification of Covariates of Interest Associated With Neonatal Volumetric MRI Measures at Birth (N=280)

eTable 2. Identification of Potential Covariates of Interest Associated With Infant Sex (N=280)

eTable 3. Comparison of Full-term Infants Excluded Due to Missing/Low-Quality MRI Data

eTable 4. Comparison of Full Cohort and Infants Excluded From (Largely Due to Prematurity) the Current Study

eTable 5. Full Results of Hierarchical Linear Regression Linking Maternal Social Disadvantage and Psychosocial Stress With Structural MRI Measures at Birth (N=280)

eTable 6. Hierarchical Linear Regression Linking Maternal Social Disadvantage and Psychosocial Stress with Total Brain Volumes (TBV) at Birth (N=280)

eTable 7. Hierarchical Linear Regression Exploring Hemispheric Effects of Maternal Social Disadvantage and Psychosocial Stress (N=280)

This supplementary material has been provided by the authors to give readers additional information about their work.

© 2022 Triplett RL et al. *JAMA Network Open.*

eFigure. Participant Flow From Study Enrollment to Inclusion in Current Analysis

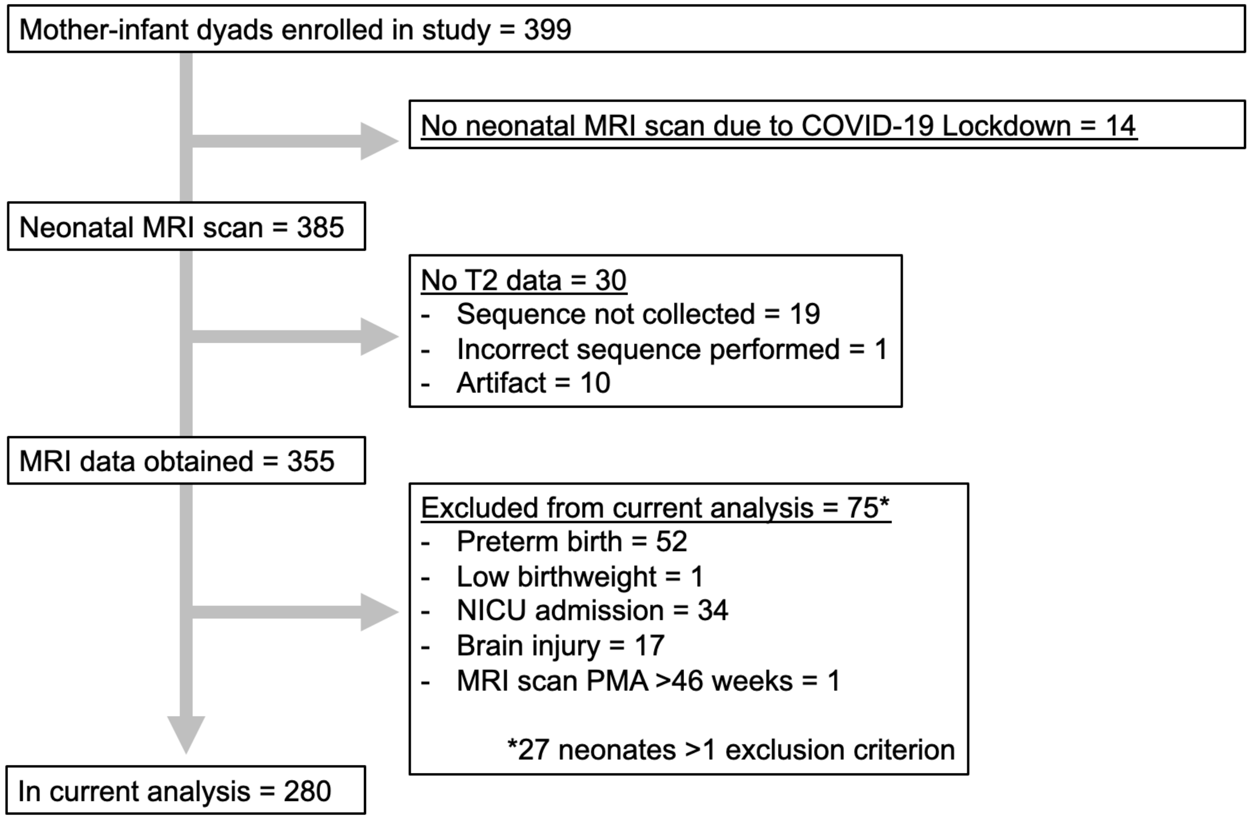

© 2022 Triplett RL et al. *JAMA Network Open.*

eMethods. Supplemental Methods

Maternal Measures in the Social Disadvantage Construct. Health insurance status (private, public/no insurance) and highest educational level were obtained at study entry (during the first trimester). Household income and composition were obtained in each of the three trimesters and generated the Income to Needs Ratio (I/R).[1] An I/R of 1.0 is equivalent to the federal poverty line. Home addresses were obtained at birth and used to calculate the national Area Deprivation Index (ADI) percentile. The ADI scores neighborhood disadvantage using US Census data regarding poverty, education, housing, and employment, with higher values indicating greater disadvantage.[2] The Diet History Questionnaire II (DHQ-II),[3] was obtained at the time of neonatal scan. The DHQ-II is a yearly food frequency measure used to characterize nutrition via the Healthy Eating Index (HEI).[4]

Maternal Measures in the Psychosocial Stress Construct. Psychological measures of the Perceived Stress Scale (PSS)[5] and the Edinburgh Postnatal Depression Scale (EPDS)[6] were collected in each trimester. The Stress and Adversity Inventory (STRAIN)[7] is a composite measure of stressful and traumatic life experiences that was obtained at time of neonatal scan (*n*=186) or at follow-up at one or two years (*n*=77). On post-hoc analyses, we did not find differences in the STRAIN stressful/traumatic life event count (*t-statistic*=.85, *two-tailed p*=0.4) or severity (*t-statistic*=1.01, *two-tailed p*=0.3) between mothers who had this collected at birth or at subsequent follow up. The Everyday Discrimination Scale (EDS) was obtained at time of neonatal scan and was scored for the "day-to-day" experience of racial discrimination, with participant response choices that ranged from "never" or "less than once a year" to "every day".[8]

Latent Constructs. Confirmatory factor analysis, distinct from exploratory factor analysis, confirms that variables identified *a priori* load on each factor. MPlus software was used to validate our *a priori* grouping of early life adversity variables into a *Social Disadvantage* latent

factor (variables listed above) and a *Psychosocial Stress* factor (variables listed above). Maximum likelihood estimation was used to derive latent factor scores for these two composite measures for all participants, despite occasional missing datapoints in observed variables.[9] Self-reported race was highly correlated with Social Disadvantage, offering no additional improvement to the model after other variables were accounted for and, thus, it was not included in either the latent Social Disadvantage or Psychosocial Stress composites. Additionally, maternal substance use, health, and BMI all have complex relationships with both SES and psychosocial factors. Therefore, we analyzed these measures independently of our defined constructs of Social Disadvantage and Psychosocial Stress.

MRI Data Collection, Preprocessing, and Brain Volumetric Measures. T1- and T2-weighted and spin echo fieldmap data were acquired with the following sequence parameters, T1: repetition time (TR)=2400ms, echo time (TE)=2.22ms, voxel size=0.8×0.8×0.8 mm^3; T2: TR=3200/4500ms, TE=563ms, tissue T2=160ms, voxel size=0.8×0.8×0.8 mm^3, and spin echo: TR=8000ms, TE=66ms, voxel size=2×2×2 mm^3; 2 mm isotropic, multiband factor (MB)=1.

The T2-weighted images were first reviewed by a highly experienced imaging scientist (D.A.) and pediatric neurologist (C.D.S.) and evaluated based on image quality and estimated subject motion. Subjects determined to have severe motion during the scan (*n*=10) were not included in subsequent analyses.

The T2-weighted images were then preprocessed using the following standard steps: gradient and readout distortion correction using the Human Connectome Project preprocessing pipeline,[10] FSL axis reorientation to the MNI152 standard-space template,[11] image denoising using Advanced Normalization Tools for Brain and Image Analysis (ANTS) Registration Suite,[12] and co-registration using the Washington University School of Medicine Neuroimaging Laboratory (NIL)'s 4-dimensional floating point (4dfp)-based image analysis.[13] The resulting T2

© 2022 Triplett RL et al. *JAMA Network Open.*

images were then used as input for Melbourne Children's Regional Infant Brain atlas Surface (M-CRIB-S) segmentation and surface extraction toolkit, which automatically generated anatomical volume segmentations and reconstructed cortical surfaces.[14,15] The M-CRIB-S toolkit included N4 bias field correction and brain extraction, as well as automatic segmentation into white and gray matter, cerebellum, brainstem, and subcortical gray matter subdivisions corresponding to FreeSurfer-like labeling. Curvature-based spherical registration and mapping, alignment, and averaging were performed, allowing for spatial normalization within the cohort and to the M-CRIB atlas.

The segmentation volumes and the cortical surfaces were then projected on the T2 images (using Connectome Workbench[16] and ITK-SNAP[17] software packages) to qualitatively evaluate the concordance between segmentations and anatomic structures (including subcortical regions) and cortical surface reconstructions and anatomic gyral and sulcal morphometry. Segmentations and surfaces were rated independently by D.A. and a second, highly experienced rater (D.M.) for necessary edits as is standard with these analysis methods.[18,19,20,21] For a subset of subjects, segmentations were then manually edited (D.A. and D.M.) using the ITK-SNAP toolkit, and surfaces were regenerated using the M-CRIB-S toolkit. Edits were performed in all three planes to ensure accurate delineation of structures, primarily the supratentorial gray matter, white matter, and cerebrospinal fluid, also with minor edits of the subcortical structures and the cerebellum. Edited segmentations and surfaces were inspected iteratively, with additional minor edits, if necessary. Final segmentations and surfaces were reviewed and designated as complete by D.A. and C.D.S.

<u>eReferences</u>

1. Brooks-Gunn J, Klebanov PK, Liaw F ruey. The learning, physical, and emotional environment of the home in the context of poverty: The infant health and development program. *Child Youth Serv Rev*. 1995;17(1-2):251-276. doi:10.1016/0190-7409(95)00011-Z

2. Kind AJH, Buckingham WR. Making Neighborhood-Disadvantage Metrics Accessible — The Neighborhood Atlas. *N Engl J Med*. 2018;378(26):2456-2458. doi:10.1056/NEJMp1802313

3. *Diet History Questionnaire*. National Institutes of Health, Epidemiology and Genomics Research Program, National Cancer Institute.; 2010.

4. Krebs-Smith SM, Pannucci TE, Subar AF, et al. Update of the Healthy Eating Index: HEI-2015. *J Acad Nutr Diet*. 2018;118(9):1591-1602. doi:10.1016/j.jand.2018.05.021

5. Cohen S, Kamarck T, Mermelstein R. A global measure of perceived stress. *J Health Soc Behav*. 1983;24(4):385-396.

6. Cox JL, Holden JM, Sagovsky R. Detection of Postnatal Depression: Development of the 10-item Edinburgh Postnatal Depression Scale. *Br J Psychiatry*. 1987;150(6):782-786. doi:10.1192/bjp.150.6.782

7. Slavich GM, Shields GS. Assessing Lifetime Stress Exposure Using the Stress and Adversity Inventory for Adults (Adult STRAIN): An Overview and Initial Validation. *Psychosom Med*. 2018;80(1):17-27. doi:10.1097/PSY.0000000000000534

8. Williams DR, Yan Yu, Jackson JS, Anderson NB. Racial Differences in Physical and Mental Health: Socio-economic Status, Stress and Discrimination. *J Health Psychol*. 1997;2(3):335-351. doi:10.1177/135910539700200305

9. Luby JL, Barch DM, Warner B, et al. *Modeling Prenatal Adversity/Advantage: Effects on Birth Weight*. MedRxiv; 2021. doi:10.1101/2021.12.16.21267938

10. Glasser MF, Sotiropoulos SN, Wilson JA, et al. The minimal preprocessing pipelines for the Human Connectome Project. *NeuroImage*. 2013;80:105-124. doi:10.1016/j.neuroimage.2013.04.127

11. Jenkinson M, Beckmann CF, Behrens TEJ, Woolrich MW, Smith SM. FSL. *NeuroImage*. 2012;62(2):782-790. doi:10.1016/j.neuroimage.2011.09.015

12. Manjón JV, Coupé P, Martí-Bonmatí L, Collins DL, Robles M. Adaptive non-local means denoising of MR images with spatially varying noise levels: Spatially Adaptive Nonlocal Denoising. *J Magn Reson Imaging*. 2010;31(1):192-203. doi:10.1002/jmri.22003

13. Snyder AZ, Acevedo H, Koller J. *4dfp Suite*. Washington University in St. Louis; 2021. https://4dfp.readthedocs.io/

14. Alexander B, Murray AL, Loh WY, et al. A new neonatal cortical and subcortical brain atlas: the Melbourne Children's Regional Infant Brain (M-CRIB) atlas. *NeuroImage*. 2017;147:841-851. doi:10.1016/j.neuroimage.2016.09.068

15. Adamson CL, Alexander B, Ball G, et al. *Parcellation of the Cortex Using Surface-Based Melbourne Children's Regional Infant Brain Atlases (M-CRIB-S)*. Neuroscience; 2019. doi:10.1101/544304

16. *Connectome Workbench*. Washington University in St. Louis; 2020. https://www.humanconnectome.org/software/connectome-workbench

© 2022 Triplett RL et al. *JAMA Network Open.*

17. Yushkevich PA, Piven J, Hazlett HC, et al. User-guided 3D active contour segmentation of anatomical structures: Significantly improved efficiency and reliability. *NeuroImage*. 2006;31(3):1116-1128. doi:10.1016/j.neuroimage.2006.01.015

18. Qiu A, Rifkin-Graboi A, Chen H, et al. Maternal anxiety and infants' hippocampal development: timing matters. *Transl Psychiatry*. 2013;3(9):e306-e306. doi:10.1038/tp.2013.79

19. Wang C, Shen M, Guillaume B, et al. FKBP5 Moderates the Association between Antenatal Maternal Depressive Symptoms and Neonatal Brain Morphology. *Neuropsychopharmacol Off Publ Am Coll Neuropsychopharmacol*. 2018;43(3):564-570. doi:10.1038/npp.2017.232

20. Lehtola SJ, Tuulari JJ, Scheinin NM, et al. Newborn amygdalar volumes are associated with maternal prenatal psychological distress in a sex-dependent way. *NeuroImage Clin*. 2020;28:102380. doi:10.1016/j.nicl.2020.102380

21. Beare RJ, Chen J, Kelly CE, et al. Neonatal Brain Tissue Classification with Morphological Adaptation and Unified Segmentation. *Front Neuroinformatics*. 2016;10. doi:10.3389/fninf.2016.00012

© 2022 Triplett RL et al. *JAMA Network Open.*

eTable 1. Identification of Covariates of Interest Associated With Neonatal Volumetric MRI Measures at Birth (N=280)

	**Maternal BMI [a]**		**MMR Score**		**Maternal Marijuana Exposure [b]**		**Maternal Tobacco Use**		**Infant Birthweight**		**Infant PMA at scan**		**Infant Sex**	
**Volume**	***r***	***p***	***r***	***p***	***t***	***p***	***t***	***p***	***r***	***p***	***r***	***p***	***t***	***p***
TBV	-.074	.28	-.036	.55	-2.06	**.04**	-3.74	**<.001**	.432	**<.001**	.519	**<.001**	6.33	**<.001**
Total cGM	-.046	.50	-.024	.69	-1.22	.23	-3.14	**.002**	.440	**<.001**	.618	**<.001**	5.48	**<.001**
Total subcortical GM	-.084	.22	-.047	.44	-1.30	.20	-3.77	**<.001**	.410	**<.001**	.618	**<.001**	5.53	**<.001**
Total WM	-.082	.23	-.038	.53	-2.54	**.01**	-3.86	**<.001**	.373	**<.001**	.307	**<.001**	6.19	**<.001**
Total Cerebellum	-.076	.27	-.039	.52	-1.41	.16	-2.69	**.008**	.372	**<.001**	.678	**<.001**	5.24	**<.001**
Gyrification Index	-.071	.30	-.035	.56	2.36	**.02**	-.64	.53	.270	**<.001**	.472	**<.001**	5.56	**<.001**

MMR = Maternal Medical Risk, PMA = postmenstrual age, TBV = total brain volume, cGM = cortical gray matter, GM = gray matter, WM = white matter. *r*=Pearson's correlation coefficient. *t*=independent samples t-test statistic. *p* values are two-tailed. Values are unadjusted.

[a]Maternal pre-pregnancy BMI data available for n=218

[b]Marijuana exposure included all mothers with a urine drug screen positive for tetrahydrocannabinol metabolites and/or self-reported marijuana use during pregnancy

© 2022 Triplett RL et al. *JAMA Network Open.*

eTable 2. Identification of Potential Covariates of Interest Associated With Infant Sex (N=280)

**Variable, mean (SD)**	**Males (n=149)**	**Females (n=131)**	***t***	***p***
Birthweight (g)	3316 (470)	3191 (500)	2.159	**.03**
PMA at Scan (weeks)	41.74 (1.27)	41.58 (1.30)	1.074	.28
Social Disadvantage	-.086 (1.01)	.004 (.921)	.786	.43
Psychosocial Stress	-.169 (.875)	.049 (.891)	-1.135	.26

© 2022 Triplett RL et al. *JAMA Network Open.*

eTable 3. Comparison of Full-term Infants Excluded Due to Missing/Low-Quality MRI Data

	**Adequate MRI (n=280)**	**Missing/Low-Quality MRI (n=27*)**	χ^2	**p**
Males, n (%)	149 (53)	16 (59)	.362	.55
Maternal Tobacco Use, n (%)	36 (13)	3 (11)	.068	.80
Maternal Marijuana Exposure, n (%)	74 (26)	5 (19)	.806	.37
			**t**	**p**
Birthweight (g), mean (SD)	3258 (488)	3274 (532)	.169	.87
PMA at Scan (weeks), mean (SD)	41.7 (1.3)	42.8 (1.8)	3.17	**.004**
Maternal MMR score, mean (SD)	1.0 (1.3)	1.0 (1.0)	-.009	.99
Social Disadvantage, mean (SD)	-.04 (.97)	.178 (.82)	1.32	.20
Psychosocial Stress, mean (SD)	-.11 (.88)	.16 (1.10)	1.55	.12

*n=3 infants met other exclusion criteria (*e.g.,* preterm, NICU stay)

eTable 4. Comparison of Full Cohort[9] and Infants Excluded From (Largely Due to Prematurity) the Current Study

	**Current sample (n=280)**	**Included in Luby *et al*., excluded from current sample (n=119)**	χ^2	**p**
Males, n (%)	149 (53)	72 (61)	1.80	.18
Maternal Tobacco Use, n (%)	36 (13)	14(12)	.091	.76
Maternal Marijuana Exposure, n (%)	74 (26)	22 (18)	2.89	.09
			**t**	**p**
EGA at birth (weeks), mean (SD)	38.6 (1.0)	36.5 (2.9)	-7.61	**<.001**
Birthweight (g), mean (SD)	3258 (488)	2845 (726)	-5.68	**<.001**
PMA at Scan (weeks), mean (SD)	41.7 (1.3)	41.4 (1.9)*	-1.49	.14
Maternal MMR score, mean (SD)	1.0 (1.3)	1.8 (2.3)	3.56	**<.001**
Social Disadvantage, mean (SD)	-.04 (.97)	.10 (.90)	1.45	.15
Psychosocial Stress, mean (SD)	-.11 (.88)	.27 (1.0)	3.58	**<.001**

*n = 105 with attempted MRI scans.

© 2022 Triplett RL et al. *JAMA Network Open.*

eTable 5. Full Results of Hierarchical Linear Regression Linking Maternal Social Disadvantage and Psychosocial Stress With Structural MRI Measures at Birth (N=280)

	Step 1		Step 2			Change Statistics	
**VOLUMES**	**β**[a]	**P**	**β**[a]	**P**	**FDR-adjusted q**	**ΔR²**	**F Δ (p)**
**Cortical Gray Matter**	R^2 = .542, p < .001		R^2 = .558, p < .001				
Sex	.231	<.001	.236	<.001	<.001		
Birthweight	.285	<.001	.244	<.001	<.001		
PMA at scan	.538	<.001	.523	<.001	<.001		
Tobacco Use	-.064	.13	-.028	.52	.69		
Disadvantage			-.130	.008	.01	.016	.007
Psychosocial Stress			-.024	.59	.64		
**Subcortical Gray Matter**	R^2 = .533, p < .001		R^2 = .559, p < .001				
Sex	.232	<.001	.237	<.001	<.001		
Birthweight	.248	<.001	.197	<.001	<.001		
PMA at scan	.542	<.001	.524	<.001	<.001		
Tobacco Use	-.105	.014	-.059	.17	.67		
Disadvantage			-.156	.002	.003	.026	<.001
Psychosocial Stress			-.046	.30	.60		
**White Matter**	R^2 = .295, p < .001		R^2 = .364, p < .001				
Sex	.280	< .001	.290	< .001	<.001		
Birthweight	.270	< .001	.184	< .001	.001		
PMA at scan	.223	< .001	.190	< .001	<.001		
Tobacco Use	-.123	.018	-.049	.34	.69		
Disadvantage			-.282	< .001	<.001	.069	< .001
Psychosocial Stress			-.025	.64	.64		
**Cerebellum**	R^2 = .570, p < .001		R^2 = .587, p < .001				
Sex	.227	< .001	.228	< .001	<.001		
Birthweight	.205	< .001	.168	< .001	<.001		
PMA at scan	.617	< .001	.605	< .001	<.001		
Tobacco Use	-.046	.26	-.011	.79	.79		
Disadvantage			-.093	.05	.05	.017	.004
Psychosocial Stress			-.077	.08	.30		
	**Step 1**		**Step 2**			**Change Statistics**	
**REGIONS OF INTEREST**	**β**[a]	**p**	**β**[a]	**p**	**FDR-adjusted q**	**ΔR²**	**F Δ (p)**
**Left Hippocampus**	R^2 = .197, p < .001		R^2 =.221, p < .001				

© 2022 Triplett RL et al. *JAMA Network Open.*

Sex	.154	.006	.161	.003	.008		
Birthweight	.160	.005	.109	.06	.06		
PMA at scan	.313	< .001	.293	< .001	<.001		
Tobacco Use	-.075	.18	-.033	.57	.70		
Disadvantage			-.177	.007	.01	.023	.019
Psychosocial Stress			.019	.75	.93		
**Right Hippocampus**	R^2 =.197, p < .001		R^2 = .220, p < .001				
Sex	.134	.02	.141	.01	.02		
Birthweight	.196	<.001	.145	.01	.02		
PMA at scan	.283	<.001	.262	<.001	<.001		
Tobacco Use	-.103	.06	-.060	.29	.58		
Disadvantage			-.176	.007	.01	.023	.02
Psychosocial Stress			.013	.82	.93		
**Left Amygdala**	R^2 = .377, p < .001		R^2= .408, p < .001				
Sex	.290	< .001	.298	<.001	<.001		
Birthweight	.184	<.001	.126	.01	.02		
PMA at scan	.393	<.001	.370	<.001	<.001		
Tobacco Use	-.133	.007	-.083	.09	.58		
Disadvantage			-.200	<.001	.003	.031	<.001
Psychosocial Stress			.005	.92	.93		
**Right Amygdala**	R^2 = .387, p < .001		R^2 = .416, p < .001				
Sex	.273	<.001	.281	<.001	<.001		
Birthweight	.228	<.001	.171	<.001	.003		
PMA at scan	.398	<.001	.375	<.001	<.001		
Tobacco Use	-.105	.03	-.056	.25	.58		
Disadvantage			-.194	<.001	.003	.029	.001
Psychosocial Stress			.005	.93	.93		
**Standardized Left Hippocampus** [b]	R^2 .010, p = .43		R^2 = .014, p = .57				
Sex	-.075	.22	-.073	.23	.31		
PMA at scan	-.055	.36	-.051	.41	.41		
Tobacco Use	.014	.82	.001	.99	.99		
Disadvantage			.017	.81	.86	.004	.58
Psychosocial Stress			.055	.41	.92		
**Standardized Right Hippocampus** [b]	R^2 = .024, p = .08		R^2 = .027, p = .19				

© 2022 Triplett RL et al. *JAMA Network Open.*

Sex	-.114	.06	-.112	.07	.11		
PMA at scan	-.101	.09	-.097	.11	.18		
Tobacco Use	-.021	.73	-.032	.61	.70		
Disadvantage			.012	.86	.86	.003	.67
Psychosocial Stress			.049	.46	.92		
**Standardized Left Amygdala** [b]	R^2 = .011, p = .40		R^2 = .020, p = .36				
Sex	.039	.52	.041	.50	.57		
PMA at scan	-.078	.20	-.066	.28	.38		
Tobacco Use	-.059	.33	-.085	.18	.58		
Disadvantage			.056	.42	.67	.009	.28
Psychosocial Stress			.062	.35	.92		
**Standardized Right Amygdala** [b]	R^2 = .004, p = .78		R^2 = .012, p = .64				
Sex	.010	.87	.013	.83	.83		
PMA at scan	-.060	.32	-.051	.41	.41		
Tobacco Use	-.020	.74	-.043	.50	.70		
Disadvantage			.040	.56	.75	.008	.32
Psychosocial Stress			.070	.29	.92		
	**Step 1**		**Step 2**		**FDR-adjusted**	**Change Statistics**	
**Gyrification Index**	**β** [a]	**p**	**β** [a]	**p**	**q**	**ΔR^2**	**F Δ (p)**
	R^2 = .264, p < .001		R^2 = .312, p < .001				
Sex	.104	.05	.115	.03	.03		
Birthweight	.170	.002	.098	.07	.07		
PMA at scan	.432	<.001	.402	<.001	<.001		
Tobacco Use	.037	.48	.097	.07	.07		
Disadvantage			-.260	<.001	<.001	.048	<.001
Psychosocial Stress			.042	.46	.46		

[a] Standardized coefficient values.
[b] Birthweight was not included as an independent variable for relative region of interest volumes adjusted for total brain volume to avoid overfitting. Standardized region of interest volumes were computed as the volume of the region divided by total brain volume.

© 2022 Triplett RL et al. *JAMA Network Open.*

eTable 6. Hierarchical Linear Regression Linking Maternal Social Disadvantage and Psychosocial Stress with Total Brain Volumes (TBV) at Birth (N=280)

	**Step 1**		**Step 2**		**Change Statistics**	
**Total Brain Volume**	**β** [a]	**p**	**β** [a]	**p**	**ΔR^2**	**F Δ (p)**
	R^2 = .468, p < .001		R^2 = .510, p < .001			
Sex	.275	< .001	.282	< .001		
Birthweight	.289	< .001	.407	< .001		
PMA at scan	.432	< .001	.221	< .001		
Tobacco Use	-.099	.029	-.040	.37		
Disadvantage			-.214	< .001	.043	< .001
Psychosocial Stress			-.034	.47		

[a] Standardized coefficient values

© 2022 Triplett RL et al. *JAMA Network Open.*

eTable 7. Hierarchical Linear Regression Exploring Hemispheric Effects of Maternal Social Disadvantage and Psychosocial Stress (N=280)

	Step 1		Step 2		Change Statistics	
**VOLUMES**	**β** [a]	**p**	**β** [a]	**p**	**ΔR^2**	**F Δ (p)**
**Left Hemispheric Cortical GM**	R^2 = .539, p < .001		R^2 = .556, p < .001			
Sex	.227	<.001	.231	<.001		
Birthweight	.280	<.001	.237	<.001		
PMA at scan	.542	<.001	.526	<.001		
Tobacco Use	-.063	.13	-.026	.54		
Disadvantage			-.136	.006	.017	.005
Psychosocial Stress			-.022	.62		
**Right Hemispheric Cortical GM**	R^2 = .538, p < .001		R^2 = .553, p < .001			
Sex	.234	<.001	.238	<.001		
Birthweight	.289	<.001	.250	<.001		
PMA at scan	.531	<.001	.516	<.001		
Tobacco Use	-.064	.13	-.029	.50		
Disadvantage			-.123	.01	.015	.01
Psychosocial Stress			-.025	.57		
**Left Hemispheric Cerebral WM**	R^2 = .291, p < .001		R^2 = .365, p < .001			
Sex	.279	<.001	.289	<.001		
Birthweight	.272	<.001	.182	<.001		
PMA at scan	.217	<.001	.183	<.001		
Tobacco Use	-.121	.02	-.043	.40		
Disadvantage			-.297	<.001	.075	<.001
Psychosocial Stress			-.020	.71		
**Right Hemispheric Cerebral WM**	R^2 = .295, p < .001		R^2 = .358, p < .001			
Sex	.280	<.001	.289	<.001		
Birthweight	.267	<.001	.185	<.001		
PMA at scan	.227	<.001	.196	<.001		
Tobacco Use	-.125	.02	-.053	.30		
Disadvantage			-.266	<.001	.063	<.001
Psychosocial Stress			-.030	.58		
**Left Cerebellum**	R^2 = .551, p < .001		R^2 = .565, p < .001			

© 2022 Triplett RL et al. *JAMA Network Open.*

Sex	.214	<.001	.213	<.001		
Birthweight	.195	<.001	.167	<.001		
PMA at scan	.612	<.001	.604	<.001		
Tobacco Use	-.052	.21	-.024	.57		
Disadvantage			-.061	.21	.014	.012
Psychosocial Stress			-.089	.05		
**Right Cerebellum**	R^2 = .566, p < .001		R^2 = .587, p < .001			
Sex	.235	<.001	.238	<.001		
Birthweight	.210	<.001	.167	<.001		
PMA at scan	.609	<.001	.594	<.001		
Tobacco Use	-.038	.35	.001	.97		
Disadvantage			-.122	.01	.021	.001
Psychosocial Stress			-.064	.14		
**Left Hemispheric GI**	R^2 = .219, p < .001		R^2 = .258, p < .001			
Sex	.057	.29	.065	.22		
Birthweight	.143	.01	.078	.17		
PMA at scan	.410	<.001	.385	<.001		
Tobacco Use	.030	.57	.085	.13		
Disadvantage			-.220	<.001	.039	<.001
Psychosocial Stress			-.001	.99		
**Right Hemispheric GI**	R^2 = .252, p < .001		R^2 = .301, p < .001			
Sex	.137	.01	.150	.004		
Birthweight	.178	.001	.107	.05		
PMA at scan	.403	<.001	.372	<.001		
Tobacco Use	.040	.45	.097	.07		
Disadvantage			-.268	<.001	.049	<.001
Psychosocial Stress			.078	.17		

[a] Standardized coefficient values

© 2022 Triplett RL et al. *JAMA Network Open.*
